# scVIC: deep generative modeling of heterogeneity for scRNA-seq data

**DOI:** 10.1093/bioadv/vbae086

**Published:** 2024-06-13

**Authors:** Jiankang Xiong, Fuzhou Gong, Liang Ma, Lin Wan

**Affiliations:** National Center for Mathematics and Interdisciplinary Sciences, Academy of Mathematics and Systems Science, Chinese Academy of Sciences, Beijing 100190, China; School of Mathematical Sciences, University of Chinese Academy of Sciences, Beijing 100049, China; National Center for Mathematics and Interdisciplinary Sciences, Academy of Mathematics and Systems Science, Chinese Academy of Sciences, Beijing 100190, China; School of Mathematical Sciences, University of Chinese Academy of Sciences, Beijing 100049, China; School of Mathematical Sciences, University of Chinese Academy of Sciences, Beijing 100049, China; Key Laboratory of Zoological Systematics and Evolution, Institute of Zoology, Chinese Academy of Sciences, Beijing 100101, China; National Center for Mathematics and Interdisciplinary Sciences, Academy of Mathematics and Systems Science, Chinese Academy of Sciences, Beijing 100190, China; School of Mathematical Sciences, University of Chinese Academy of Sciences, Beijing 100049, China

## Abstract

**Motivation:**

Single-cell RNA sequencing (scRNA-seq) has become a valuable tool for studying cellular heterogeneity. However, the analysis of scRNA-seq data is challenging because of inherent noise and technical variability. Existing methods often struggle to simultaneously explore heterogeneity across cells, handle dropout events, and account for batch effects. These drawbacks call for a robust and comprehensive method that can address these challenges and provide accurate insights into heterogeneity at the single-cell level.

**Results:**

In this study, we introduce scVIC, an algorithm designed to account for variational inference, while simultaneously handling biological heterogeneity and batch effects at the single-cell level. scVIC explicitly models both biological heterogeneity and technical variability to learn cellular heterogeneity in a manner free from dropout events and the bias of batch effects. By leveraging variational inference, we provide a robust framework for inferring the parameters of scVIC. To test the performance of scVIC, we employed both simulated and biological scRNA-seq datasets, either including, or not, batch effects. scVIC was found to outperform other approaches because of its superior clustering ability and circumvention of the batch effects problem.

**Availability and implementation:**

The code of scVIC and replication for this study are available at https://github.com/HiBearME/scVIC/tree/v1.0.

## 1 Introduction

Cells are fundamental units of multicellular organisms, and each cell plays a unique biological role in growth and development. In large part, the heterogeneity among cells arises from differences in the expression of genetic information, such as transcriptome; therefore, the analysis of differential expression becomes an important step in determining the role of single cells. Recently, there has been an upswing in the development of tools aimed at the analysis of high-throughput, single-cell RNA sequencing data (scRNA-seq data). scRNA-seq allows the collection of massive transcriptional expression at single-cell resolution, making it an ideal tool to study the cell–cell heterogeneity in developmental biology, oncology, and immunology. However, technical difficulties have arisen because of inefficient RNA capture and the requirement for deep sequencing to capture low-abundance transcripts in single cells, which, however, can cause frequent dropout events, making transcriptional expression noisy and sparse (Hebenstreit 2012). Another technical variability involves the bias of batch effects, which has received increased attention as transcriptome sources become more diverse. However, the introduction of batch effects can also confound biological heterogeneity (Hicks *et al.* 2018). Failure to eliminate these two technical influences will inevitably complicate subsequent analysis and lead to misinterpretation of the resultant heterogeneity.

An essential step in exploring cellular heterogeneity is the ability to cluster cells into subpopulations. To date, numerous clustering methods for scRNA-seq data have been proposed. To handle dropout events, CIDR (clustering through imputation and dimensionality reduction) first imputes gene expression profiles and then performs hierarchical clustering on its main coordinates of dissimilarity matrix through principal coordinate analysis (Lin *et al.* 2017). SIMLR (Single-cell Interpretation *via* Multi-kernel LeaRning) first learns a similarity measure from single-cell RNA-seq data *via* multiple kernel learning, which applies a designed diffusion-based step to reduce the effects of noise and dropout events. Spectral clustering is then performed on the similarity measure (Wang *et al.* 2017). Rare cell type identification (RaceID) was developed to reveal rare cell types in complex populations of single cells, basically by *k*-means clustering and, recently, an improved version by *k*-medoids clustering on the similarity matrix (Grün *et al.* 2016). Semisoft clustering with pure cells (SOUP) is an algorithm that reveals the clustering structure for both pure and transitional cells. It first identifies pure cells from an expression similarity matrix and then applies *k*-means clustering to the pure cells (Zhu *et al.* 2019). However, neither RaceID nor SOUP explicitly models the technical variability that results from dropout events and then learns a similarity representation based on the original noisy data matrix, which is no longer accurate. Moreover, these four methods separate similarity representation from subsequent clustering, which often leads to spurious heterogeneity. Under the recently popular framework of Neural Networks (NN), scDeepCluster provides a deep count autoencoder algorithm (Eraslan *et al.* 2019) that nonlinearly reduces the high-dimensionality of original data to the low-dimensionality of a latent space while reconstructing the denoised data. This is combined with a deep embedding clustering algorithm (Xie *et al.* 2016) designed to simultaneously apply clustering on compressed data residing in latent space (Tian *et al.* 2019). scziDesk improves upon scDeepCluster in that it learns a more cluster-friendly latent space by gradually strengthening the affinity within highly similar compressed data (Chen *et al.* 2020). Recently, graph neural networks have also been successfully applied to the unsupervised clustering of scRNA-seq data (Wang *et al.* 2021, Gu *et al.* 2022). Lei *et al.* (2023) specifically discussed the above methods and concluded that they all have room for improvement.

Apart from dropout events, scRNA-seq is challenged by data that may originate from multiple batches. Absent proper accounting, batch effects can be misinterpreted as true biological signals. The widespread application of scRNA-seq only means new computational challenges for integrating datasets from different batches and platforms as more complex datasets are presented (Yu *et al.* 2023). The clustering algorithms mentioned above do not consider the bias caused by batch effects. Indeed, for datasets with strong batch effects, the authors of these algorithms recommend that researchers first use other existing algorithms to remove batch effects. For example, to address batch effects, the single-cell data analysis tool Seurat (Stuart *et al.* 2019) first exploits canonical correlation analysis (CCA) (Butler *et al.* 2018) to analyze the correlation between two batches of single-cell transcriptome data. Then, it further exploits the mutual nearest neighbor (MNN) (Haghverdi *et al.* 2018) to match cells between the two batches. Based on neural networks, scVI (single-cell variational inference) (Lopez *et al.* 2018) integrates measured expression values to approximate the true posterior distribution of variables in the latent space, while simultaneously considering the capture of bias caused by dropout events and batch effects.

However, the algorithms mentioned above typically only target either batch effects removal or clustering. For example, scVI does not model cell heterogeneity in a built-in manner. To further explore heterogeneity, scVI suggests using other classical clustering algorithms on inferred latent variables. To establish a heterogeneous model, the Gaussian Mixture Variational Autoencoder (GMVAE) in scVAE incorporates a categorical latent random variable into the traditional probabilistic graph for single-cell gene expression data in the context of variational autoencoder (VAE) (Grønbech *et al.* 2020). It uses two additional neural networks to deduce the assumed conditional prior distribution given different categories and variational approximate conditional posterior distribution given measured expression values. However, GMVAE does not consider batch effects. For simultaneous batch effects removal and clustering of scRNA-seq data analysis, specific clustering methods have been proposed. For instance, deep embedding for single-cell clustering (DESC) removes batch effects in single-cell analysis by employing neural networks to iteratively optimize clustering objective functions on scRNA-seq data. As long as the technical variability between batches is smaller than the true biological heterogeneity, DESC can gradually eliminate batch effects (Li *et al.* 2020). scDEC exploits generative adversarial networks (GANs) to simultaneously learn the latent representation and infer cell labels, which could be extended for the integrative analysis of multi-batch scRNA-seq data (Liu *et al.* 2021). Single-cell multi-omics deep clustering (scMDC) employs a multimodal autoencoder to jointly learn the latent features of deep embedding for clustering analysis across different data sources (Jovic *et al.* 2022).

Herein, we introduce deep generative modeling of heterogeneity for scRNA-seq data (scVIC), which accommodates Gaussian mixture modules (GMM) under the framework of VAE. We also propose a specific coordinate descent algorithm to optimize all parameters, including those in VAE and GMM, to make the training robust. scVIC explicitly models both biological heterogeneity and technical variability derived from dropout events and the bias of batch effects; therefore, inferred heterogeneity is free from technical variability.

## 2 Methods

### 2.1 scVIC

**Figure 1.**
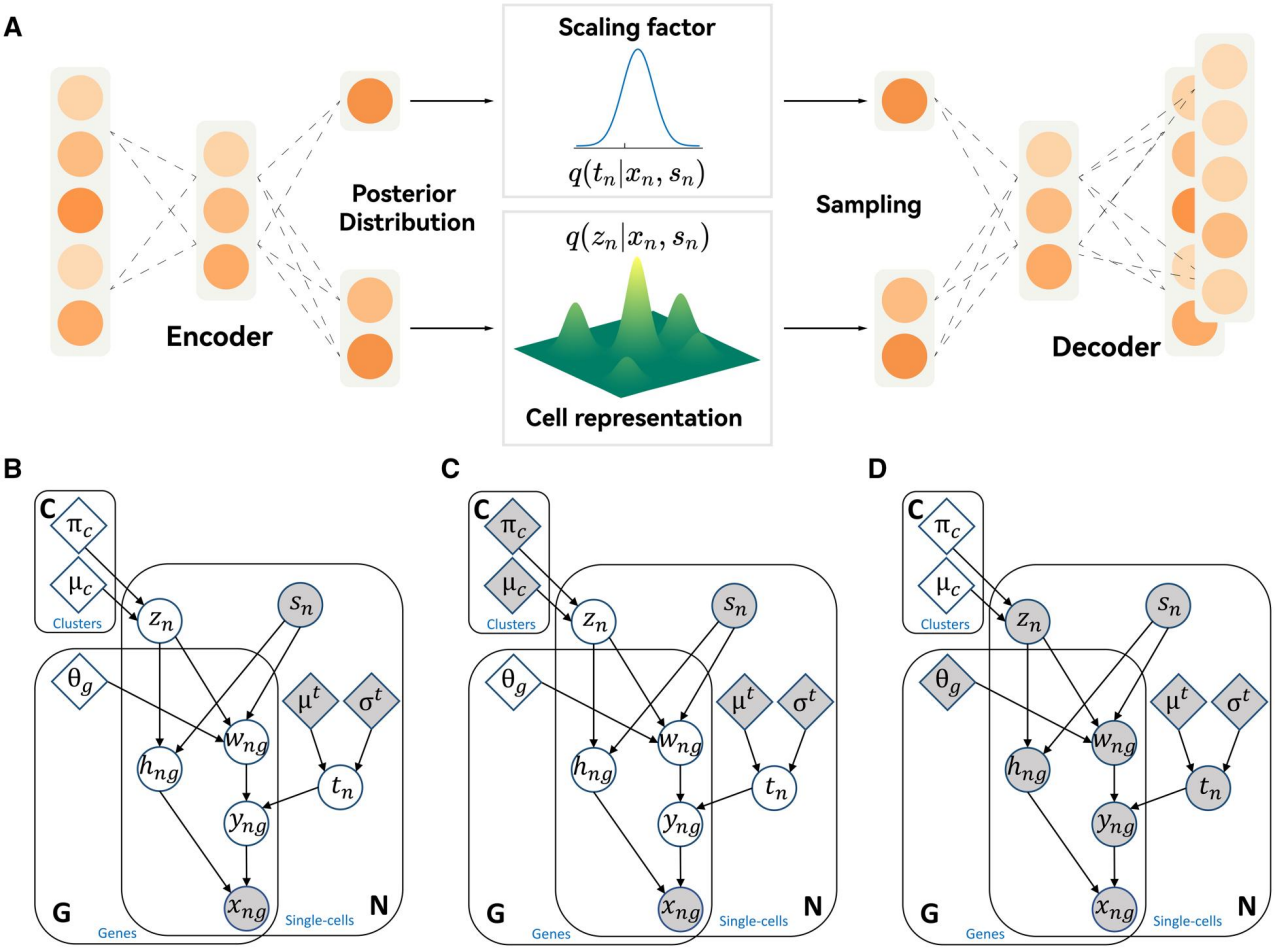
Overview of scVIC. (A) Neural network schematic of scVIC. (B) Generative probabilistic model of scVIC. Shaded vertices represent observed random variables, and empty vertices represent unobserved latent random variables. Shaded diamonds represent constants as a priori, and empty diamonds represent global parameters that need to be estimated. Edges signify conditional dependency. (C) Probabilistic model when parameters of Gaussian mixture distribution are fixed during parametric optimization. (D) Probabilistic model when parameters of the NN are fixed during parametric optimization.

We assume that the differential expression of single cells originates from some latent variables that exhibit heterogeneity, and with that assumption, we proceed to construct a neural network to learn the generative process from these latent variables to the posterior distribution of gene expression. The neural network architecture of scVIC within the framework of a VAE is schematically illustrated in Fig. 1A. The probabilistic decoder on the right corresponds to the generative process of single-cell gene expression, which is triggered by two latent variables through probabilistic decoding. These two unmeasured latent variables refer to the scaling factor and intrinsic cell representation, respectively. The scaling factor represents technical variability caused, e.g. by capture efficiency and sequencing depth. Intrinsic cell representation, i.e. a vector representation of the inherent properties of single cells, is assumed to be a biological factor underlying differential expression among cells. Both factors are assumed to follow Gaussian-based distributions such that the scaling factor is assumed to be a 1D normal distribution, while whereas representation is assumed to be a mixture of Gaussian distributions, thus describing the inherent biological heterogeneity of single cells. Using the probabilistic decoder corresponding to these two factors, we can obtain specific parametric values of gene expression distribution, and we specify gene expression distribution as the classical zero-inflated negative binomial (ZINB) distribution. The probabilistic encoder on the left corresponds to the inference of the conditional (variational) posterior distribution of two latent variables given the measured gene expression. The measured gene expression is fed into the neural network corresponding to the probability encoder to obtain the specific distribution parameters of the assumed variational posterior distribution. This variational posterior distribution is used to approximate the true posterior distribution of the two latent variables. If the expression comes from different experimental batches, batch effects removal is necessary. Therefore, we also fed the batch annotation into the neural network to model the batch effects.

The optimization of scVIC consists of two parts. One part is conducted under the framework of variational inference and stochastic optimization, while the other part is conducted under the framework of expectation–maximization (EM). Together, these two parts form our final optimization algorithm based on coordinate descent.

### 2.2 Probabilistic model

First, we present the assumed probabilistic generative process of single-cell gene expression in the scVIC model, along with the corresponding probabilistic graph model shown in Fig. 1B. Each observed expression value *x_ng_* is independently drawn from the following process:

(1) $$z_{n}∼\text{GaussianMixture}({\left[\pi_{c}\right]}_{C},{\left[\mu_{c}\right]}_{C}),$$

(2) $$t_{n}∼\text{Normal}({\bar{\mu }}^{t},{\bar{\sigma }}^{t}{\;}^{2}),$$

(3) $$\rho_{n}=f_{\rho |z,s}(z_{n},s_{n}),$$

(4) $$w_{ng}∼\text{Gamma}(\rho_{n}^{g},\theta_{g}),$$

(5) $$y_{ng}∼\text{Possion}(t_{n}w_{ng}),$$

(6) $$\alpha_{n}=f_{\alpha |z,s}(z_{n},s_{n}),$$

(7) $$h_{ng}∼\text{Bernoulli}(\alpha_{n}^{g}),$$

(8) $$x_{ng}=\{\begin{array}{cc} y_{ng}, & h_{ng}=0\\ 0, & \text{otherwise}. \end{array}$$

Equation (1) models the probability of latent variables corresponding to the intrinsic biological properties of single cells. Equation (2) models the probability of latent variables corresponding to single-cell scaling factors, and Equations (3)–(8) represent ZINB distribution involving scaling factors, which is modeled as a probabilistic neural network that serves as the recognition model in VAE. By iteratively mapping and sampling through the neural networks, *x_ng_* can be generated from two sampled latent variables, which is actually a probabilistic encoding process in the semantic context of variational autoencoders. In Equations (3) and (6), the variable *s_n_* refers to the additional measured information variables of the nth cell, such as batch annotation, and these are also fed into the two neural networks. The variable *h_ng_* in Equation (7) is a binary variable constructed by simulating whether the nth cell drops out the gth gene expression in the ZINB distribution. By incorporating these two variables, the probabilistic generative model assumed by scVIC successfully and effectively models the technical variability of dropout events and batch effects.

In common VAE, the prior distribution for the latent variable *z* is set to standard multivariate Gaussian (Kingma and Welling 2014). To incorporate heterogeneity, we let *z* be Gaussian mixture, while the covariance of each mixture component remains the identity matrix. *C* denotes the number of mixture components of GMM, and over the cth component, *π_c_* denotes mixture ratio and *μ_c_* denotes mean of GMM; therefore, the density function of the simplified GMM is calculated as:

$$p(z)=\sum_{c=1}^{C}\pi_{c}N(z;\mu_{c},I).$$

Our notation basically follows that from scVI (Lopez *et al.* 2018). $l_{\mu }$ and $l_{\sigma }\in R_{+}^{B}$ in Equation (2) parameterize prior distribution for the scaling factor, where *B* denotes the number of batches, and ${\bar{\mu }}^{t}$ and ${\bar{\sigma }}^{t}$ are set to be the empirical mean and variance of the log-library size per batch, respectively. The parameter $\theta_{g}\in R_{+}$ in Equation (4) denotes gene-specific inverse dispersion, and $\rho_{n}^{g}$ denotes mean of the negative binomial distribution in the ZINB distribution. $f_{\rho |z,s}$ in Equation (3) and $f_{\alpha |z,s}$ in Equation (6) are neural networks mapping the latent space and batch annotation to the dimension of genes: $R^{d}\times {\left\{0,1\right\}}^{B}\to R^{G}$. To avoid ambiguity, the learnable parameters of the neural network have been omitted in the notation. Superscript annotation ($\rho_{n}^{g},\alpha_{n}^{g}$) refers to a single entry corresponding to a specific gene *g*. Network $f_{\rho |z,s}$ decodes mean of proportional transcription expression across genes, and the sum of the mean over the gene dimension equals 1. The intermediate vector $t_{n}w_{ng}$ can therefore be interpreted as the expected transcriptional expression of gene *g*. Network $f_{\alpha |z,s}$ decodes the frequency of technical dropout events occurring at a particular entry (Pierson and Yau 2015).

### 2.3 Inference *via* coordinate descent

Given the prior generative process of *x_ng_* and the observed transcriptional expression matrix *X*, all we want are posterior inference. However, the true posterior density is intractable in our case because the denominator (integral of the marginal distribution over all the latent variables) $p(x_{n}|s_{n})$ is intractable in the Bayes rule. Note that the latent variables *w_ng_*, *h_ng_*, and *y_ng_* can be integrated out because $p(x|z_{n},l_{n},s_{n})$ has a closed-form density, which is actually a ZINB distribution (Grün *et al.* 2014). The zero-inflated negative binomial distribution based on a neural network with a scaling factor is denoted as the ZINB-NN distribution (see details in Supplementary Note 1). The ZINB-NN contains three parameters: the mean of the scaled negative binomial distribution $\mu_{n}^{g}=t_{n}f_{\rho |z,s}^{g}(z_{n},s_{n})$, the probability of zero inflation $\alpha_{n}^{g}=f_{\alpha |z,s}^{g}(z_{n},s_{n})$, and the inverse dispersion parameter for each gene *θ_g_*.

Then, variational inference (Blei *et al.* 2017) is exploited to approximate the posterior $p(z_{n},l_{n}|x_{n},s_{n})$ (see details in Supplementary Note 2). Based on the mean-field assumption, the variational lower bound is calculated as follows:

$$\begin{array}{c} L=\sum_{n}E_{q(z,t|x_{n},s_{n})}\;\text{log}\;p(x_{n}|z,t,s_{n})\\ -\sum_{n}KL(q(t|x_{n},s_{n})||p(t))\\ -\sum_{n}E_{q(z|x_{n},s_{n})}[\text{log}\;q(z|x_{n},s_{n})]\\ \;\;\;+\sum_{n}E_{q(z|x_{n},s_{n})}[\text{log}\;p(z)]\\ \leq \sum_{n}\;\text{log}\;p(x_{n}|s_{n}). \end{array}$$

In this objective function, we divide the trainable parameters into two parts. The first part consists of the dispersion parameter *θ_g_* for each gene and the neural network parameters in $f_{\rho |z,s},\;f_{\alpha |z,s},\;f_{[\mu^{z},\sigma^{z}]|x,s}$ and $f_{[\mu^{t},\sigma^{t}]|x,s}$. The second part consists of parameters of *μ_c_* and *π_c_*. When the parameters of the second part are fixed, the parameters of the first part are optimized directly in a variational Bayesian inference fashion, and the specific probabilistic graph is shown in Fig. 1C. Conversely, when the parameters of the first part are fixed, the parameters of second part can be optimized within the framework of EM, and the specific probabilistic graph is shown in Fig. 1D. Therefore, coordinate descent on two blocks is exploited to optimize all trainable parameters.

When the parameters of the second part are fixed, we further unfold the GMM by an additional categorical latent random variable as:

(9) $$c_{n}∼\text{Categorical}({\left[\pi_{c}\right]}_{C}),$$

(10) $$z_{n}∼\text{Normal}(\mu_{c_{n}},I).$$

Equation (9) denotes category distribution for the component latent variable, and Equation (10) denotes the normal distribution corresponding to each category. Then, we obtain another variational lower bound:

$$\begin{array}{c} L\prime =\sum_{n}E_{q(z,t|x_{n},s_{n})}\;\text{log}\;p(x_{n}|z,t,s_{n})\\ \;\;\;\;-\sum_{n}KL(q(t|x_{n},s_{n})||p(t))\\ \;\;\;\;-\sum_{n}E_{q(z|x_{n},s_{n})}[\text{log}\;q(z|x_{n},s_{n})]\\ \;\;\;\;-\sum_{n}E_{q(z|x_{n},s_{n})}[KL(q(c|z)||p(c))]\\ \;\;\;\;+\sum_{n}E_{q(z|x_{n},s_{n})}[\sum_{c=1}^{C}q(c|z)\;\text{log}\;p(z|c)]\\ \leq \sum_{n}\;\text{log}\;p(x_{n}|s_{n}). \end{array}$$

When the variation distribution approximates with sufficient gradation to the posterior distribution, both can converge to the marginal likelihood. Therefore, we optimize the lower bound $L^{\prime }$. The specific form of $p(x_{n}|z,t,s_{n})$ is given by Supplementary Equation (S1). The assumptions for $q(z,t|x_{n},s_{n}),\;q(z|x_{n},s_{n})$, and $q(t|x_{n},s_{n})$ are given by Supplementary Equations (S2), (S3), and (S4), respectively. *p*(*t*) is given by Equation (2). *p*(*c*) and p(z|c) are given by Equations (9) and (10). The closed form of $KL(q(t|x_{n},s_{n})||p(t))$ (see details in Supplementary Note 3) is given by Supplementary Equation (S5).

The distribution corresponding to the categorical variable *c* is a discrete categorical distribution, so $KL(q(c|z_{n})||p(c))$ can be solved by directly summing over the categories, and the closed form of q(c|z) is given as:

$$q(c|z)=\frac{\pi_{c}N(z;\mu_{c},I)}{\sum_{c}\pi_{c}N(z;\mu_{c},I)}.$$

Then, the parameters of the first part can be optimized in the framework of variational inference and stochastic optimization, using the reparameterization and mini-batch trick employed in VAE. After computing the gradient of the variational lower bound $L^{\prime }$ with respect to the first set of parameters using the backpropagation algorithm, we use the gradient-based optimizer Adam to update these parameters. This optimization is based on a neural network framework, which naturally allows parallel computation.

When the parameters of the first part are fixed, minimization of the variational lower bound L equals maximization by

$$L\prime \prime =\sum_{n}E_{q(z|x_{n},s_{n})}[\text{log}\;p(z)].$$

Since Monte Carlo sampling is completed upon optimization of the parameters of the first part, maximizing L″ equals maximum likelihood estimation of GMM on the Monte Carlo samples drawn from the variational approximate posterior of the latent variable *z*, which can then be completed by classic EM. Specifically, in the ith optimization iteration, given the lth sampling point of nth cell and cth component, the estimation for step *i* is

$$q_{c|z}^{\left(i\right)}(z_{n}^{l})=\frac{\pi_{c}^{\left(i-1\right)}N(z_{n}^{l};\mu_{c}^{\left(i-1\right)},I)}{\sum_{c}\pi_{c}^{\left(i-1\right)}N(z_{n}^{l};\mu_{c}^{\left(i-1\right)},I)}.$$

Correspondingly, the maximization of EM, which actually optimizes the parameters in the Gaussian mixture, for step *i* is

$$\begin{array}{c} \mu_{c}^{\left(i\right)}=\frac{\sum_{n}\sum_{l}q_{c|z}^{\left(i\right)}(z_{n}^{l})z_{n}^{l}}{\sum_{n}\sum_{l}q_{c|z}^{\left(i\right)}(z_{n}^{l})},\\ \pi_{c}^{\left(i\right)}=\frac{\sum_{n}\sum_{l}q_{c|z}^{\left(i\right)}(z_{n}^{l})}{N\times L}. \end{array}$$

EM is carried out on the low-dimensional space of the latent variable *z_n_*, in turn making it easy to perform parallel computations.

We integrate the two optimization steps and use iterative coordinate descent to infer all parameters of scVIC. Algorithm 1 provides a detailed description.

Hyper-parameters of scVIC are set to default values of scVI, where the dimensionality of *z* is set to 10, the number of samples *L* is 1, the training batch size *N_b_* is 128, the maximum number of epochs *N_e_* is 400, and the learning rate is 1e−3.

Algorithm 1. Coordinate descent of scVIC

**Require:** Expression matrix ${\left[x_{n}\right]}_{N}$, Batch annotation ${\left[s_{n}\right]}_{N}$, Number of batches *N_b_*, Number of epochs *N_e_*, Four initialized neural networks, and Initialized distribution parameters from ZINB and GM.

**Ensure:** Four optimized neural networks and Optimized distribution parameters from ZINB and GM.

 **Initialize** neural networks.

 **Initialize**${\left[\theta_{g}\right]}_{G},{\left[\pi_{c}\right]}_{C}$ and ${\left[\mu_{c}\right]}_{C}$.

 **for** $e\in [1,N_{e}]$ **do**

  **for** $b\in [1,N_{b}]$ **do**

   **for** $n\in {\text{Batch}}_{b}$ **do**

     $\left[\mu_{n}^{z},\sigma_{n}^{z}]=f_{[\mu^{z},\sigma^{z}]|x,s}(x_{n},s_{n}\right),$

     $\left[\mu_{n}^{t},\sigma_{n}^{t}]=f_{[\mu^{t},\sigma^{t}]|x,s}(x_{n},s_{n}\right).$

▹ Probabilistic Encoder.

    **for** l∈[1,L]**do**

     $z_{n}^{l}∼\text{Gaussian}(\mu_{n}^{z},\sigma_{n}^{z}{\;}^{2}I),$

     $t_{n}^{l}∼\text{LogNormal}(\mu_{n}^{t},\sigma_{n}^{t}{\;}^{2})$. ▹ Sampling.

     **for** c∈[1,C] **do**

       $q_{c|z}(z_{n}^{l})=\frac{\pi_{c}N(z_{n}^{l};\mu_{c},I)}{\sum_{c}\pi_{c}N(z_{n}^{l};\mu_{c},I)}.$

▹ Expectation of EM.

     **end** **for**

    **end** **for**

   **end** **for**

$\begin{array}{c} \tilde{L\prime }=\sum_{n\in {\text{Batch}}_{b}}(-KL(q(t|x_{n},s_{n})||p(t))\\ +\sum_{l=1}^{L}(\text{log}\;p(x_{n}|z_{n}^{l},l_{n},s_{n})\\ -\text{log}\;q(z_{n}^{l}|x_{n},s_{n})-KL(q(c|z_{n}^{l})||p(c))\\ +\sum_{c=1}^{C}(q(c|z_{n}^{l})\;\text{log}\;p(z_{n}^{l}|c)))). \end{array}$

Estimator of L′.

  **Maximize**$\tilde{L\prime }$.

**end** **for**

**for** c∈[1,C] **do**

  $\mu_{c}\leftarrow \frac{\sum_{n}\sum_{l}q_{c|z}(z_{n}^{l})z_{n}^{l}}{\sum_{n}\sum_{l}q_{c|z}(z_{n}^{l})},$

  $\pi_{c}\leftarrow \frac{\sum_{n}\sum_{l}q_{c|z}(z_{n}^{l})}{N\times L}$. ▹ Maximization of EM.

**end** **for**

**if** $\tilde{L\prime }$ converges **then**

   **break**

 **end** **if**

**end** **for**

### 2.4 Clustering

After parametric optimization based on coordinate descent is completed, the transcriptomic expression *x_n_* can be probabilistically encoded through the learned neural network using latent variables. The distribution parameters of one latent variable, *z_n_*, are determined by the trained neural network $f_{[\mu^{z},\sigma^{z}]|x,s}$; therefore, the expectation $\mu_{n}^{z}$ can be regarded as an encoded form of *x_n_* in a deterministic manner. Then, in the probabilities computed by $q_{c|z}(\mu_{n}^{z})$, the category corresponding to the maximum value can be assigned as the labeled category of *x_n_*. This clustering process is a built-in part of scVIC and does not require the use of other clustering algorithms. This, however, does not preclude the use of other clustering algorithms to enhance the deterministic encoding of $\mu_{n}^{z}$. When compared with other dimensionality reduction and clustering algorithms, clustering on scVIC latent space can improve clustering performance essentially because the latent variables obtained by scVIC consider heterogeneity. We chose the popular algorithm of community detection, Louvain (Blondel *et al.* 2008), as the default clustering algorithm. Therefore, to distinguish the source of labels, we use “scVIC-Louvain” to denote the application of the Louvain algorithm to the encoding of scVIC.

### 2.5 Datasets

We evaluated scVIC using five real biological scRNA-seq datasets and one simulated dataset. We selected datasets whose size was >10^4^, including three datasets without batch effects [TRACHEA, TURTLE, and BACH, referred to Chen *et al.* (2020)] and two datasets with batches [PBMC and RETINA, referred to Lopez *et al.* (2018)].

The TRACHEA dataset (Schaum *et al.* 2018) consists of gene expression data from 11 269 mouse tracheal tissue cells obtained using 10× genomics sequencing technology. The dataset includes annotations for five biological cell types provided by the original authors.

The TURTLE dataset (Tosches *et al.* 2018) consists of gene expression data from 18 664 cells in the dorsal cortex of turtle. The dataset includes biological annotations for the 15 cell types provided by the original authors.

The BACH dataset (Bach *et al.* 2017) consists of gene expression data from 23 184 epithelial cells. The dataset includes annotations for eight major biological cell types provided by the original authors.

The PBMC dataset consists of scRNA-seq data from two batches of peripheral blood mononuclear cells (PBMCs) from a healthy donor (4K PBMCs and 8K PBMCs) (Zheng *et al.* 2017). Quality control metrics were derived using the R package cellrangerRkit (v.1.1.0). Quality metrics were extracted from CellRanger throughout the molecule-specific information file. After filtering as in Cole *et al.* (2019), 12 039 cells are extracted with 10 310 sampled genes, and then is used to obtain biologically meaningful clusters (Macosko *et al.* 2015). Genes that could not be matched with the bulk data used for differential expression are filtered, were which we were left with g=3346.

The RETINA dataset consists of bipolar cells from Shekhar *et al.* (2016); it contains 27 499 cells and 13 166 genes from two batches after their original pipeline for filtering. Biological cluster annotation from 15 cell types from the author was used.

In the simulation, we first used an R package called “splatter” (Zappia *et al.* 2017) to generate simulation datasets. Our experiments can be divided into two parts: one that does not consider the bias of batch effects and one that consider the bias of batch effects. For the first type of simulated data, we compare scVIC, which does not consider the bias of batch effects, with many methods that are not equipped to remove batch effects. To make this comparison, we further design two different simulation scenarios, one involving cell clusters of the same size and the other involving cell clusters of different sizes, which we, respectively call “balanced experiments” and “imbalanced experiments.” In the balanced experiments, we generated datasets with clusters 3, 4, 5, 6, and 7. In the imbalanced experiments, we generated datasets fixed at five clusters, but the cluster group size followed a geometric sequence with ratio ranges in {0.6, 0.7, 0.8, 0.9, 1.0}, where smaller ratio means a larger difference in cluster size. For both, we set a dropout rate that ranges from 5% to 25% (dropout.type = “experiment”, dropout.shape = −1, de.facScale = 0.2 and dropout.mid ranges from −1.5 to 0.5, while other parameters are set to default values) to simulate the “dropout” event. Both the gene number and cell number are fixed at 2500. For every scenario, we generate 10 datasets with different random seed to inspect the average performance of the different methods by calculating their median ARI and NMI values. For the second type of simulated data, we consider that real transcriptome expression data may come from different batches; therefore, we included batch effects in the simulation. Similarly, we designed two different scenarios for balanced and imbalanced simulations with parameter settings identical to those without batch effects. Here, for the simulation of batch effects, we fixed the dataset to three batches, the size of which follows a geometric sequence with a ratio of 0.7.

### 2.6 Evaluation metrics

#### 2.6.1 Clustering metrics: adjusted rand index

Given the two clustering results *U* and *V*, we calculate the following four quantities: (a) number of objects in a pair placed in the same group in *U* and in the same group in *V*; (b)number of objects in a pair placed in the same group in *U* and in different groups in *V*; (c) number of objects in a pair placed in the same group in *V* and in different groups in *U*; and (d) number of objects in a pair placed in different groups in *U* and in different groups in *V*. Adjusted rand index(ARI) has been proposed in the form of

$$\mathrm{ARI}=\frac{\left(\begin{array}{c} n\\ 2 \end{array}\right)(a+d)-[(a+b)(a+c)+(c+d)(b+d)]}{\left(\begin{array}{c} n\\ 2 \end{array}\right)-[(a+b)(a+c)+(c+d)(b+d)]}.$$

#### 2.6.2 Clustering metrics: normalized mutual information

Given two clustering results *U* and *V* on a set of data points, NMI is defined as I(U,V)/max(H(U),H(V)), where *I*(*U*, *V*) is the mutual information between *U* and *V*, and *H*(*U*) represents the entropy of the clustering result *U*

$$I(U,V)=\sum_{p=1}^{P}\sum_{q=1}^{Q}\frac{U_{p}\cap V_{q}}{N}\;\text{log}\;\frac{N|U_{p}\cap V_{q}|}{\left|U_{p}|\times |V_{q}\right|},$$

where $\left|U_{p}\right|$ and $\left|V_{q}\right|$ denote the cardinality of the pth cluster in *U* and the nqth cluster in *V*, respectively. The entropy of each cluster assignment is calculated as follows:

$$\begin{array}{c} H(U)=-\sum_{p=1}^{P}\frac{\left|U_{p}\right|}{N}\;\text{log}\;\frac{\left|U_{p}\right|}{N},\\ H(V)=-\sum_{q=1}^{Q}\frac{\left|V_{q}\right|}{N}\;\text{log}\;\frac{\left|V_{q}\right|}{N}. \end{array}$$

#### 2.6.3 Divergence of batch mixing

We use KL divergence to evaluate the performance of various single-cell clustering algorithms for batch effects removal, i.e. the stochasticity of cells from different batches mixed together within each cluster. The KL divergence of batch mixing for *B* different batches is calculated as

$$KL=\sum_{b=1}^{B}q_{b}\;\text{log}\;\frac{q_{b}}{p_{b}},$$

where *p_b_* is the proportion of cells from batch *b* among all cells, and *q_b_* is the proportion of cells from batch *b* in a given region based on clustering algorithm results. We calculate the KL divergence of batch mixing using the regional mixing KL divergence defined above using 50 randomly chosen cells from all batches. The regional proportion of cells from each batch is calculated based on the set of *K* nearest neighbor (KNN) cells from each randomly chosen cell (*K* = 50). The final KL divergence is then calculated as the average of the regional KL divergence. We repeated this procedure for 100 iterations with different randomly chosen cells and finally obtained the average.

## 3 Results

### 3.1 Heterogeneity of the simulation data

**Figure 2.**
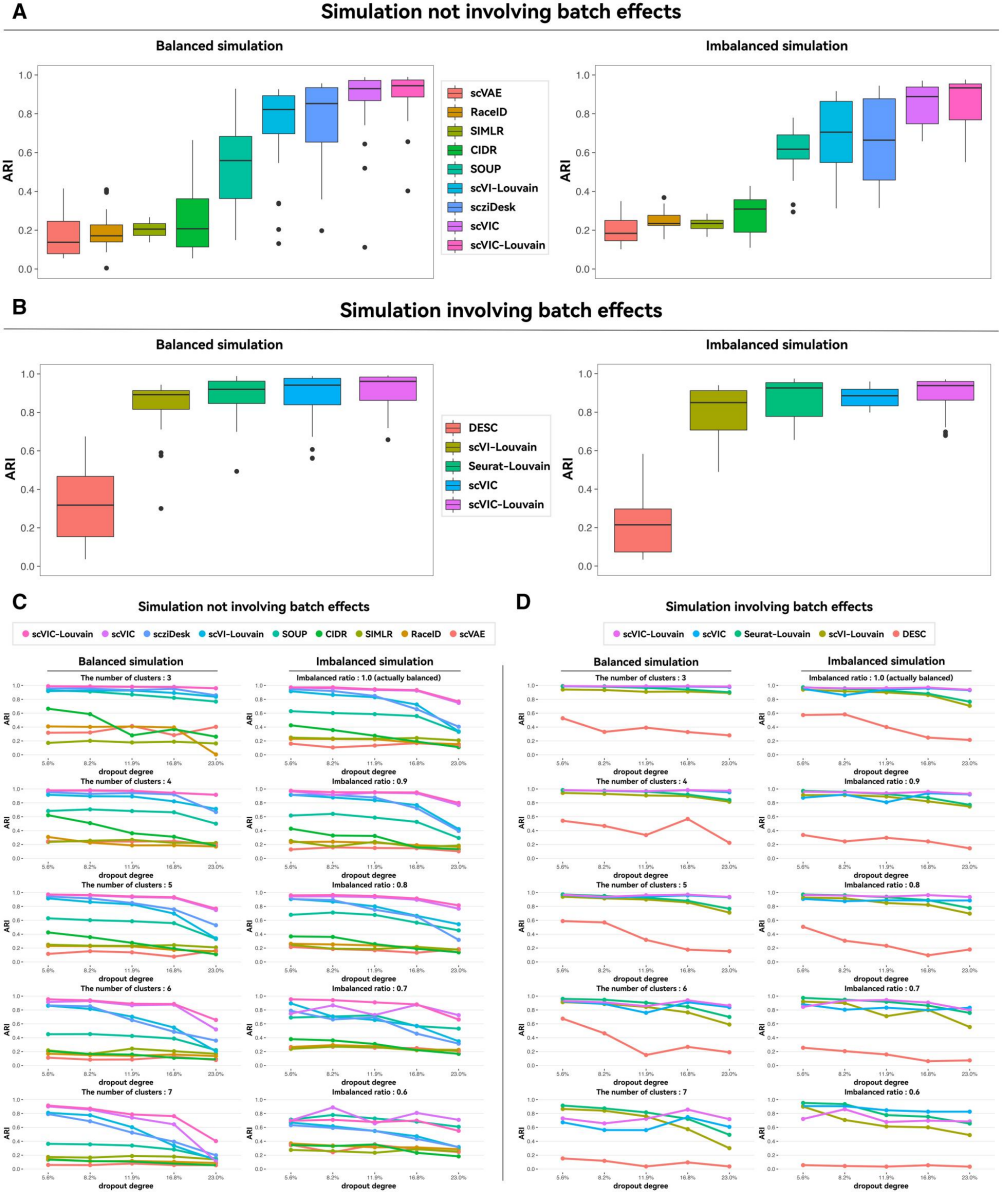
Comparison of clustering algorithms on simulation datasets. Bar charts of ARI for different clustering algorithms on simulation datasets (A) not involving batch effects and (B) involving batch effects. For different simulation parameters, line charts of ARI for different clustering algorithms on simulation datasets (C) not involving batch effects and (D) involving batch effects. The source data used in this figure is placed in the Supplementary Tables.

First, we compared the performance of all eight clustering algorithms on balanced and imbalanced simulated data without considering batch effects. As shown in Fig. 2A, scVIC achieved ARI scores of 0.93 and 0.89 on the balanced and imbalanced simulated data, respectively, while the performance of the scVIC-Louvain further improved ARI evaluation scores to 0.94 and 0.93, respectively, both significantly outperforming CIDR (0.21, 0.31), SOUP (0.56, 0.62), SIMLR (0.21, 0.23), and RaceID (0.17, 0.23). For other neural network-based algorithms, scziDesk, which also combines dimensionality reduction and clustering, achieved ARI evaluation scores of 0.85 and 0.66, respectively. Because scVI does not have built-in clustering, we similarly applied Louvain on its dimensionality reduction results, termed as scVI-Louvain, and achieved ARI evaluation scores of 0.82 and 0.71 using this simple two-step approach of dimensionality reduction and clustering. These scores are lower than those of scVIC, which is also a variational model, but considers heterogeneity. As another neural network model that considers heterogeneity, the GMVAE in scVAE achieves ARI evaluation scores of only 0.18 and 0.14, owing to its instability, even performing worse than the two-step scVI-Louvain. Our algorithm outperforms the seven other algorithms on both balanced and imbalanced simulated data without considering batch effects, as well as on NMI evaluation scores, as shown in Supplementary Fig. S1A.

As shown in Fig. 2C, the performance of the different algorithms under different simulation parameters can be further compared. Each subplot shows the performance of different algorithms as the simulated dropout rate gradually increases. As the dropout rate increases, the ARI of all algorithms decreases. However, it is noteworthy that scVIC and scVIC-Louvain are superior to other algorithms in almost all simulation parameter combinations. Furthermore, our algorithm is relatively robust, with almost all ARIs above 0.8, when the simulated data are relatively clear. For example, this occurs in balanced simulations where the number of simulated categories is ≤6, and the capture loss rate is ≤16.8%. This can also occur in imbalanced simulations where the imbalance ratio is ≤0.7, and the capture loss rate is ≤16.8%. Compared with the three other neural network-based algorithms, scziDesk and scVI-Louvain are in the second tier. The performance of scziDesk is better than that of the two-step algorithm based on the dimensionality reduction results of scVI, whereas scVAE is not comparable with other algorithms based on its instability. In the imbalanced simulated data, we noticed that SOUP, which is based on the characteristics of pure cell clustering, can effectively alleviate the difficulties caused by data imbalance, and its results are relatively stable. However, overall, it is not as good as our algorithm in terms of ARI. Its performance is only comparable to ours in the simulated data with an imbalanced ratio of 0.6. Similar results can also be obtained from the NMI evaluation metric, as shown in Supplementary Fig. S1C.

Next, we compared the performance of all four algorithms based on clustering and batch effects removal performance for both balanced and imbalanced simulated data, while considering batch effects. As shown in Fig. 2B, scVIC-Louvain has the best clustering performance on both balanced and imbalanced simulated data with ARI evaluation metrics of 0.96 and 0.94, respectively. We compared scVIC-Louvain with Seurat-Louvain and scVI-Louvain, which apply Louvain to the dimensionality reduction results obtained after batch effects removal using Seurat or scVI, respectively. scVIC and Seurat-Louvain are comparable, whereas scVI-Louvain is inferior. On balanced and imbalanced simulated data, the ARI scores for scVIC are 0.94 and 0.89, respectively, whereas for Seurat-Louvain it is 0.92 and 0.93. The ARI scores for scVI-Louvain are 0.89 and 0.85, respectively. However, it is worth noting that Seurat-Louvain performs much worse than the other algorithms in terms of batch effects removal, as shown in Supplementary Fig. S2. DESC, which is also based on neural networks and combines batch effects removal and clustering, performs poorly with ARI scores of 0.32 and 0.21, respectively. Our algorithm outperforms the other three algorithms in terms of both batch effects removal and clustering on balanced and imbalanced simulated data considering batch effects. This conclusion was also validated by the NMI score, as shown in Supplementary Fig. S1B.

The performance of different algorithms on different simulation parameters can be further compared, as shown in Fig. 2D. In most cases, scVIC and scVIC-Louvain outperform the other algorithms. Unlike the results presented in the simulation data without considering batch effects, when the simulated data are relatively clear, our algorithm is comparable to other algorithms. However, our algorithm outperforms other algorithms when the dropout rate of simulation data is relatively high, reaching up to 23%. When the simulated data are relatively clear, scVI-Louvain, as a more direct approach, can also achieve good results. Seurat-Louvain trades off poor performance in batch effects removal for good clustering results. scVIC simultaneously considers batch effects removal and clustering, especially when the simulated data have a high dropout rate. scVIC can achieve better overall results. It is worth noting that DESC, which also combines batch effects removal and clustering based on neural networks, consistently performs poorly in various parameter combinations. Similar results can also be obtained using the NMI evaluation metric, as shown in Supplementary Fig. S1D.

### 3.2 Heterogeneity of the real biological data not involving batch effects

**Figure 3.**
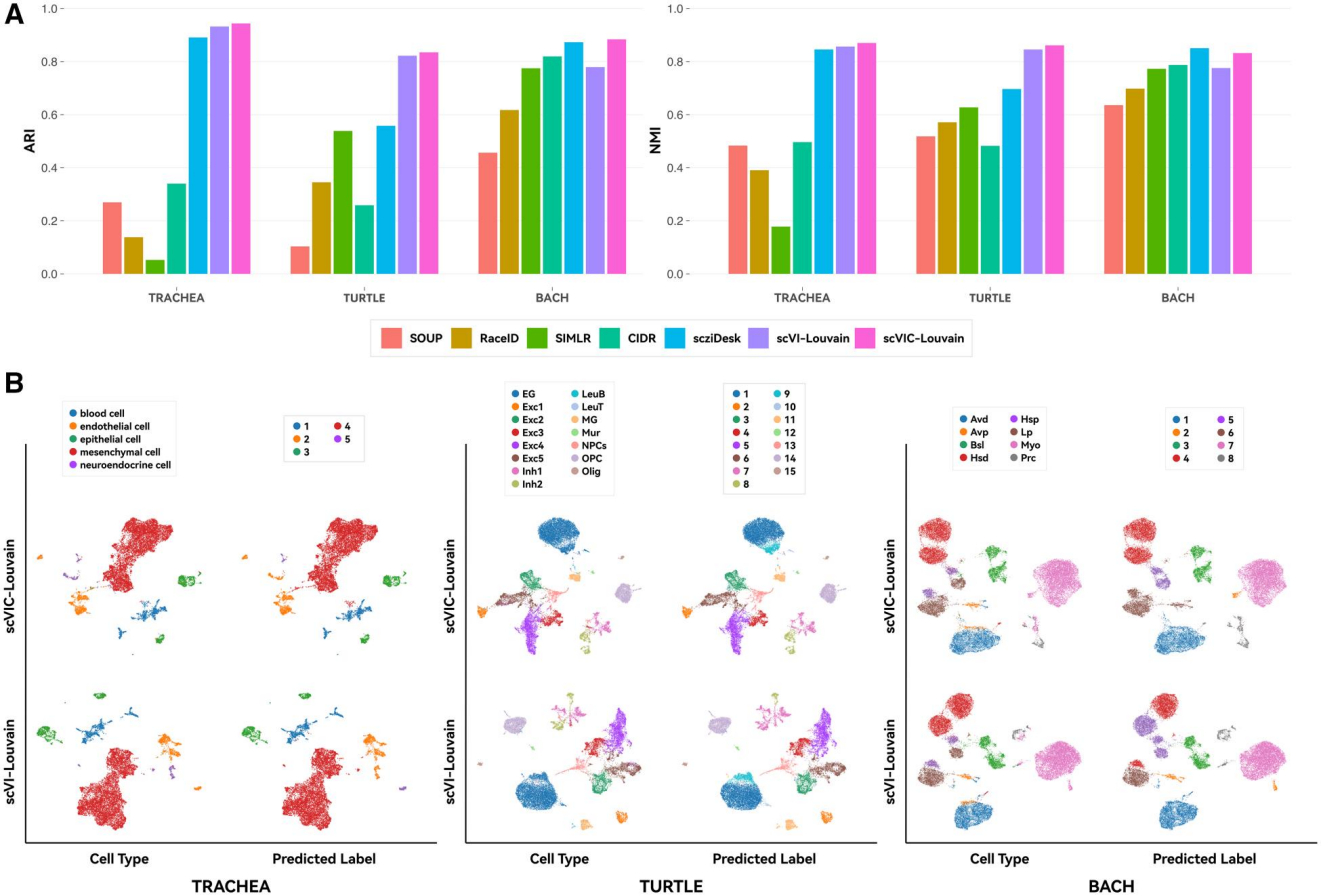
Comparison of clustering algorithms on biological datasets not involving batch effects. (A) Bar charts of clustering metrics for different algorithms. The source data used in this sub-figure is placed in the Supplementary Tables. (B) Visualization for different algorithms (scVIC-Louvain and scVI-Louvain).

We compared the performance of all seven clustering algorithms on three biological datasets, TRACHEA, TURTLE, and BACH, which were free from batch effects. It is worth mentioning that the annotation of actual biological datasets is mostly manually annotated on the basis of the results of community detection algorithms similar to Louvain combined with a certain biological significance. Therefore, we selected the results of scVIC-Louvain for comparison with those of other algorithms. In addition, based on the instability of scVAE during optimization, it was not included in the comparison below. The performance of different algorithms for both ARI and NMI evaluation metrics is shown in Fig. 3A. On the TRACHEA dataset, the performance of three neural network-based algorithms, scVIC-Louvain (with ARI and NMI values of 0.94 and 0.87, respectively), scVI-Louvain (0.93 and 0.86), and scziDesk (0.89 and 0.85), outperformed the other four algorithms, including CIDR (0.34 and 0.50), RaceID (0.14 and 0.39), SIMLR (0.05 and 0.18), and SOUP (0.27 and 0.48). On the TURTLE dataset, scVIC-Louvain (0.83 and 0.86) and scVI-Louvain (0.82 and 0.85) outperformed scziDesk (0.56 and 0.70), CIDR (0.26 and 0.48), RaceID (0.35 and 0.57), SIMLR (0.54 and 0.63), and SOUP (0.10 and 0.52). On the largest dataset, BACH, scVIC-Louvain (0.88 and 0.83), and scziDesk (0.87 and 0.85) performed similarly and slightly outperformed scVI-Louvain (0.78 and 0.78). As the data size reaches a certain scale, the performance of CIDR (0.82 and 0.79), RaceID (0.62 and 0.70), SIMLR (0.77 and 0.77), and SOUP (0.46 and 0.64) improves.

Overall, in biological datasets without batch effects, the results of scVIC-Louvain are generally better and more stable than those of scVI-Louvain and scziDesk. In turn, their performance is generally better than that of CIDR, RaceID, SIMLR, and SOUP. These four algorithms tend to perform better as the data size reaches a certain scale.

To compare their dimensionality reduction clustering results, we further projected the latent variables of scVIC and scVI onto a 2D space using the UMAP visualization algorithm (Becht *et al.* 2019) as shown in Fig. 3B. For the TRACHEA and TURTLE datasets, the clustering evaluation metrics indicate that scVIC-Louvain performs slightly better than scVI-Louvain, however there is small difference between the visualization results of the two methods. This suggests that for biological datasets on which scVI yields a strongly heterogeneous latent space, scVIC can effectively preserve similar heterogeneity. For the BACH dataset, scVI-Louvain performed significantly worse than scVIC-Louvain in clustering evaluation metrics. We can observe specific differences between the two methods in the visualization of their latent spaces, mainly concentrated in the *Hsd*, *Hsp*, and *Lp* categories. When using Louvain for further classification on the latent space generated by scVI, these three categories appear to be intertwined, while scVIC has, to some extent, mitigated this phenomenon. Using Louvain on the latent space generated by scVIC accurately identifies the class with the largest number of samples, *Hsd*. However, an overlap still exists between the *Hsp* and *Lp* classes. This phenomenon could be explained by the presence of further subdivisions of subgroups within the BACH dataset that still exhibit some degree of heterogeneity. The original author’s annotation is detailed enough to classify up to 15 categories. Within the *Hsd* category, three subcategories are possible, two for *Hsp*, and two for *Lp*. This, to some extent, validates the rationality of the two algorithms. If only following the default eight major cell types provided by the original author, scVIC outperforms scVI in terms of clustering.

### 3.3 Heterogeneity of the real biological data involving batch effects

**Figure 4.**
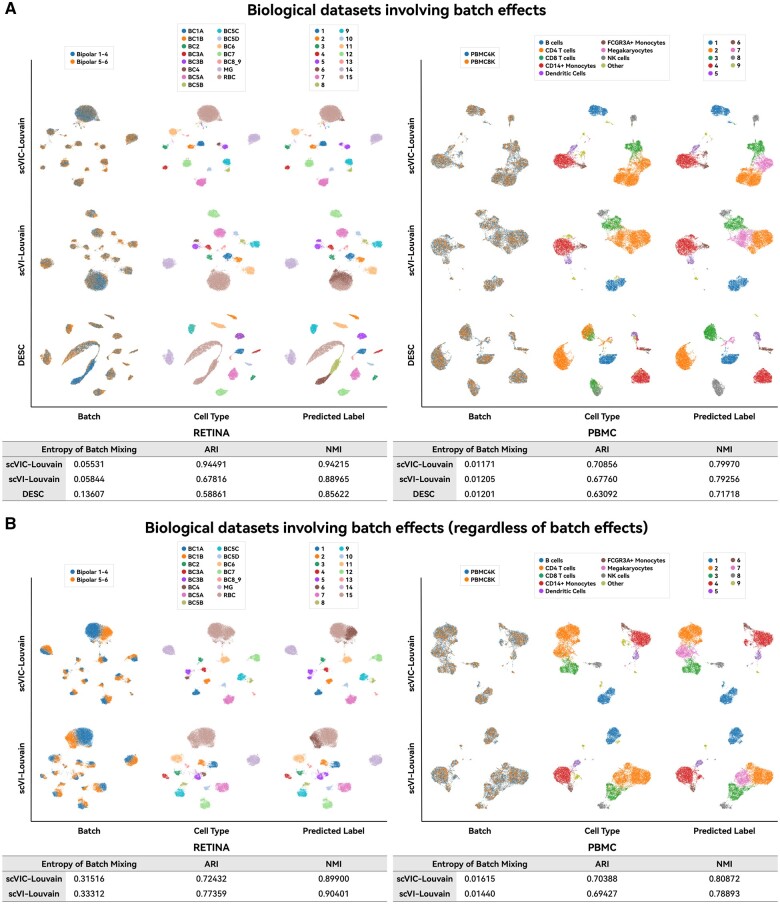
Comparison of clustering algorithms that consider batch effects on biological datasets involving batch effects. (A) Comparison of visualization performance and metrics for different algorithms that consider batch effects. (B) Comparison of visualization performance and metrics for different algorithms that do not consider batch effects (scVIC-Louvain and scVI-Louvain).

We compared three neural network-based algorithms on the biological datasets RETINA and PBMC that have batch effects, as shown in Fig. 4A. For both datasets, scVIC-Louvain outperforms scVI-Louvain and DESC, where batch effects removal is evaluated by KL distance metric. A smaller KL distance indicates better removal. For the RETINA dataset, scVIC-Louvain outperforms the other two algorithms in terms of the cluster evaluation metric ARI. From the visual results, we can see that DESC divides the largest category, *RBC*, into three subcategories, while scVI-Louvain divides it into two relatively even subcategories. However, scVIC-Louvain performed much better, and most samples were clustered into one category, which significantly improved the ARI evaluation metric. The performance of scVIC-Louvain is also superior to those of the other two algorithms based on the NMI evaluation metric that incorporates subset separation. At the same time, we can see that DESC is clearly inferior to the other two algorithms in removing batch effects, especially for the category *RBC* wherein mixture of the two batches is quite uneven, and the unevenness is clearly visible in the visualization results. We further visualize the impact of batch effects removal using different algorithms, focusing on scVIC-Louvain and scVI-Louvain. Although we know that the RETINA dataset comes from two batches, we do not input batch labels into these two algorithms, i.e. we do not consider batch effects. The visualization results obtained are shown in Fig. 4B. Both algorithms show visible splitting of each cluster into two batches, indicating that if batch effects are not considered in processing the RETINA dataset, then the resulting visualization will be heavily influenced by batch effects. The KL divergence score for batch effects increased sharply from around 0.055 to around 0.315. Such low-dimensional visualization results are unreliable. On the PBMC dataset, in addition to outperforming the other two algorithms in the clustering metric ARI, scVIC-Louvain shows results comparable to the other two evaluation metrics, particularly in comparison with scVI-Louvain. Despite two annotated batches, batch effects were sufficiently removed after preprocessing by the original authors. This can be observed in the results of the PBMC data in Fig. 4B. Therefore, using scVIC-Louvain or scVI-Louvain without considering batch annotation information yields visualization results comparable to those using algorithms with batch annotation information.

In addition to evaluating the performance of scVIC, we further compared scVIC with Harmony (Korsunsky *et al.* 2019) and LIGER (Lu and Welch 2022), the other two methods specifically designed and recommended for batch effects removal (Tran *et al.* 2020), on the RETINA dataset, which suffers from batch effects. As shown in Supplementary Fig. S3, scVIC demonstrates superior performance compared to Harmony and LIGER regarding clustering based on low-dimensional variables. This observation suggests that scVIC effectively captures the inherent biological heterogeneity present in the RNA-seq datasets. In terms of batch effects removal, scVIC’s performance is satisfactory, surpassing Harmony whereas exhibiting a slight disadvantage compared to LIGER. This outcome is understandable given that LIGER and Harmony are specifically designed to address batch effects.

## 4 Discussion and conclusion

We developed a deep generative model, scVIC, specifically designed to address the inherent biological heterogeneity in single-cell RNA sequencing of gene expression data.

scVIC is a variational autoencoder model designed for single-cell RNA sequencing of gene expression data. This variational autoencoder follows a specified probabilistic generative process in which the high-dimensional gene expression profiles of each cell can be generated from low-dimensional latent variables based on certain probabilistic assumptions. scVIC follows a neural network-based, scaling factor-free zero-inflated negative binomial generative process and approximates the posterior distribution of the latent variables based on high-dimensional gene expression as a decomposable form, whereas the variational posterior distribution parameters of the low-dimensional latent variables are inferred by the neural network. To model intrinsic cellular heterogeneity in single-cell organisms, scVIC uses a mixture of Gaussian distributions in the probabilistic generation process instead of the original standard Gaussian distribution used in the scVI model, which does not consider heterogeneity. As a result, the inferred latent space of scVIC better captures the inherent biological heterogeneity among single-cell RNA sequencing gene expression data compared with the heterogeneity-agnostic generative model scVI.

Because scVIC introduced a mixture of Gaussian distribution, it also introduced some new parameters that need to be inferred. The results of scVAE show that direct use of the categorical latent random variable from GMM as the latent variable of the model, may amplify the inferential load on the variational autoencoders, leading to inadequate inference of GMM. For the scVIC model, we derive two variational lower bounds that can converge to the log-likelihood function, and we propose a coordinate descent-based parameter training algorithm to optimize all parameters, including those in VAE and GMM. This makes the training process more robust.scVIC models the biological heterogeneity inherent in single-cell sequencing gene expression data; therefore, it can directly infer cell type classifications for different cells. It also models the technical uncertainty caused by dropout and batch effects, so the inferred heterogeneity is not biased by these two technical factors and does not require preprocessing with existing imputation algorithms or batch effects removal algorithms for single-cell sequencing gene expression data. After comparing various algorithms on both simulated and real biological datasets, scVIC performed among the top in clustering and batch effects removal evaluations.

Because scVIC is based on an interpretable probabilistic generative process, some intermediate quantities can be extracted to construct variables with specific biological meanings, e.g. the expected gene expression level after correcting for technical variability, which includes dropout events, batch effects, and sequencing depth (see details in Supplementary Note 4). More importantly, the inferred heterogeneity includes not only the clustering results, but also further differential expression, which is also built-in scVIC. We constructed a Bayesian factor to measure the degree of differential gene expression between a specified category and the rest (see methods in Supplementary Note 5 and results in Supplementary Note 6).

In short, scVIC models not only the technical variability caused by dropout events and batch effects in single-cell RNA sequencing gene expression data but also the biological heterogeneity inherent in the data. To make the training more robust, we propose a coordinate descent-based parameter training algorithm to optimize all parameters in scVIC.

## Supplementary Material

vbae086_Supplementary_Data

## Data Availability

The code of scVIC and replication for this study are available at https://github.com/HiBearME/scVIC/tree/v1.0. The preprocessed TRACHEA, TURTLE, and BACH datasets refer at https://github.com/xuebaliang/scziDesk and the preprocessed PBMC and RETINA datasets refer at https://github.com/romain-lopez/scVI-reproducibility.
