# Supplementary material for: scVIC: deep generative modeling of heterogeneity for scRNA-seq data
Source: Bioinform Adv. 2024 Jun 13;4(1):vbae086. doi: 10.1093/bioadv/vbae086 (PMC11256938; doi:10.1093/bioadv/vbae086)
Supplement: vbae086_Supplementary_Data [file vbae086_supplementary_data.zip › SupplementaryMaterial-scVIC.pdf]

scVIC: Deep generative modeling of heterogeneity for scRNA-seq data

Supplementary Information

Jiankang Xiong[1,3] Fuzhou Gong[1,3] Liang Ma[2,3,*]
Lin Wan[1,3,*]

[1]NCMIS, Academy of Mathematics and Systems Science,
Chinese Academy of Sciences, Beijing 100190, China
[2]Institute of Zoology, Chinese Academy of Sciences, Beijing 100101, China
[3]University of Chinese Academy of Sciences, Beijing 100049, China

*To whom correspondence may be addressed. The authors wish it to be known that, in their opinion, the last two authors should be regarded as Joint Corresponding Authors. Email: maliang@ioz.ac.cn (Liang Ma) and lwan@amss.ac.cn (Lin Wan).

Contents

Supplementary Method **3**

Supplementary Note 1: ZINB based on NN with scaling factor 3

Supplementary Note 2: The variational approximate distributions assumed for posterior 5

Supplementary Note 3: Closed form of KL divergence of latent variable t 6

Supplementary Note 4: Alignment on technical variability 8

Supplementary Note 5: Differential expression 10

Supplementary Note 6: Differential expression on the RETINA dataset 11

Supplementary Figures **13**

Supplementary Method

Supplementary Note 1: ZINB based on NN with scaling factor

The probabilistic generating process of the negative binomial distribution with scale factor is as follows:

$$w_n \sim \text{Gamma}(\frac{p}{1-p}, r),$$
$$y_n \sim \text{Possion}(\lambda w_n),$$

where $\lambda \in \mathbb{R}^+$ denotes scaling factor. In Gamma distributed random variable w, r denotes the shape parameters, and $\frac{p}{1-p}$ represents the scale parameter. Then the integer-valued random variable y follows a negative binomial distribution with mean $\lambda r \frac{p}{1-p}$, and its probability mass function is as follows:

$$\begin{aligned}
p(y) &= \int p(y|w)p(w)\mathrm{d}w \\
&= \int \frac{w^{r-1}e^{-w(\frac{1}{p}-1)}(1-p)^r}{p^r\Gamma(r)} \frac{e^{-\lambda w}\lambda^y w^y}{\Gamma(y+1)}\mathrm{d}w \\
&= \frac{\Gamma(y+r)}{\Gamma(y+1)\Gamma(r)}\left(\frac{1-p}{1-p+\lambda p}\right)^r \left(\frac{\lambda p}{1-p+\lambda p}\right)^y.
\end{aligned}$$

By substituting variables in the scVIC model so that scale factor $\lambda = t_n$, shape parameter of the Gamma distribution $r = \theta_g$ and mean $\frac{p}{1-p}r = \rho_n^g = f^g_{\rho|z,s}(z_n, s_n)$, we have $p = \frac{\rho_n^g}{\rho_n^g+\theta_g}$, which can be substituted into $p(y)$ as follows:

$$\begin{aligned}
p(y) &= \frac{\Gamma(y+\theta_g)}{\Gamma(y+1)\Gamma(\theta_g)}\left(\frac{1-\frac{\rho_n^g}{\rho_n^g+\theta_g}}{1-\frac{\rho_n^g}{\rho_n^g+\theta_g}+t_n\frac{\rho_n^g}{\rho_n^g+\theta_g}}\right)^{\theta_g}\left(\frac{t_n\frac{\rho_n^g}{\rho_n^g+\theta_g}}{1-\frac{\rho_n^g}{\rho_n^g+\theta_g}+t_n\frac{\rho_n^g}{\rho_n^g+\theta_g}}\right)^y \\
&= \frac{\Gamma(y+\theta_g)}{\Gamma(y+1)\Gamma(\theta_g)}\left(\frac{\theta_g}{\theta_g+t_n\rho_n^g}\right)^{\theta_g}\left(\frac{t_n\rho^g}{\theta_g+t_n\rho_n^g}\right)^y.
\end{aligned}$$

Let $\mu_n^g = t_n\rho_n^g$, then

$$p(y) = \frac{\Gamma(y+\theta_g)}{\Gamma(y+1)\Gamma(\theta_g)}\left(\frac{\theta_g}{\theta_g+\mu_n^g}\right)^{\theta_g}\left(\frac{\mu_n^g}{\theta_g+\mu_n^g}\right)^y.$$

This probability mass function corresponds to a negative binomial distribution with pa-

rameters mean μ_n^g and inverse dispersion θ_g.

In zero-inflated negative binomial (ZINB) distribution, 'zero-inflated' refers to mixing a point mass at zero with the original negative binomial distribution. Therefore, in scVIC, the probability mass function of the ZINB-NN distribution based on a neural network with scale factor is given by $p(x|z_n, t_n, s_n)$, as follows:

$$\begin{cases} p(x_g = 0|z_n, t_n, s_n) = \alpha_n^g + (1 - \alpha_n^g)(\frac{\theta_g}{\theta_g + \mu_n^g})^{\theta_g} \\ p(x_g = y|z_n, t_n, s_n) = (1 - \alpha_n^g)\frac{\Gamma(y+\theta_g)}{\Gamma(y+1)\Gamma(\theta_g)}(\frac{\theta_g}{\theta_g + \mu_n^g})^{\theta_g}(\frac{\mu_n^g}{\theta_g + \mu_n^g})^y, \forall y \in \mathbb{N}^*. \end{cases} \tag{S1}$$

This distribution includes three parameters: mean of the scaled negative binomial distribution $\mu_n^g = t_n f_{\rho|z,s}^g(z_n, s_n)$, probability of zero inflation $\alpha_n^g = f_{\alpha|z,s}^g(z_n, s_n)$ and the inverse dispersion parameter for each gene θ_g.

Supplementary Note 2: The variational approximate distributions assumed for posterior

Variational inference is exploited to approximate the posterior $p(z_n, l_n|x_n, s_n)$. We let variational approximate posterior $q(z_n, l_n|x_n, s_n)$ be calculated according to mean-field theory as

$$q(z, t|x_n, s_n) = q(z|x_n, s_n)q(t|x_n, s_n). \tag{S2}$$

The variational approximate distribution $q(z|x_n, s_n)$ is chosen to be multivariate Gaussian with a diagonal covariance matrix, mean and covariance of which are inferred through the encoder network $f_{[\mu^z,\sigma^z]|x,s}$. The probability density function based on neural networks is calculated as

$$q(z|x_n, s_n) = \prod_{i=1}^{I_z} \frac{e^{-\frac{(z[i]-\mu_n^z[i])^2}{2\sigma_n^{z\,2}[i]}}}{\sqrt{2\pi}\sigma_n^z[i]}, \tag{S3}$$

where I_z represents the dimension of latent variable z, $[i]$ represents the $i-$th component of a vector, and $[\mu_n^z, \sigma_n^z] = f_{[\mu^z,\sigma^z]|x,s}(x_n, s_n)$.

The variational approximate distribution $q(t|x_n, s_n)$ is chosen to be one-dimensional normal distribution, the scalar mean and variance of which are inferred through a second encoder network, $f_{[\mu^t,\sigma^t]|x,s}$. The probability density function based on neural networks is calculated as

$$q(t|x_n, s_n) = \frac{e^{-\frac{(t-\mu_n^t)^2}{2\sigma_n^{t\,2}}}}{\sqrt{2\pi}\sigma_n^t}, \tag{S4}$$

where $[\mu_n^t, \sigma_n^t] = f_{[\mu^t,\sigma^t]|x,s}(x_n, s_n)$.

Supplementary Note 3: Closed form of KL divergence of latent variable t

The two distributions are respectively $q(t|x_n, s_n)$ and $p(t)$. To avoid ambiguity, we remove some superscripts that do not affect the results and define them as

$$q(t|x_n, s_n) = \frac{e^{-\frac{(t-\mu_n)^2}{2\sigma_n{}^2}}}{\sqrt{2\pi}\sigma_n},$$
$$p(t) = \frac{e^{-\frac{(t-\overline{\mu})^2}{2\overline{\sigma}^2}}}{\sqrt{2\pi}\overline{\sigma}}.$$

In the definition of $q(t|x_n, s_n)$, $[\mu_n, \sigma_n] = f_{[\mu,\sigma]|x,s}(x_n, s_n)$, which is computed by a neural network. In the definition of $p(t)$, $\overline{\mu}$ and $\overline{\sigma}$ are constant values defined as prior.
$\mathbb{E}[t^2|x_n, s_n] = \mathbb{E}[t|x_n, s_n]^2 + \mathbf{Var}[t|x_n, s_n]$; therefore,

$$\int q(t|x_n, s_n)t^2\mathrm{d}t = \mu_n^2 + \sigma_n^2.$$

Combine:

$$\int q(t|x_n, s_n)t\mathrm{d}t = \mathbb{E}[t|x_n, s_n] = \mu_n.$$

Then:

$$\begin{aligned}
\int q(t|x_n, s_n)\log p(t)\mathrm{d}t &= \int q(t|x_n, s_n)\log\frac{e^{-\frac{(t-\overline{\mu})^2}{2\overline{\sigma}^2}}}{\sqrt{2\pi}\overline{\sigma}}\mathrm{d}t \\
&= -\frac{1}{2}\log(2\pi\overline{\sigma}^2) - \int q(t|x_n, s_n)\frac{(t-\overline{\mu})^2}{2\overline{\sigma}^2}\mathrm{d}t \\
&= -\frac{1}{2}\log(2\pi\overline{\sigma}^2) - \int q(t|x_n, s_n)\frac{t^2}{2\overline{\sigma}^2}\mathrm{d}t \\
&\quad + \int q(t|x_n, s_n)\frac{2t\overline{\mu}}{2\overline{\sigma}^2}\mathrm{d}t - \int q(t|x_n, s_n)\frac{\overline{\mu}^2}{2\overline{\sigma}^2}\mathrm{d}t \\
&= -\frac{1}{2}\log(2\pi\overline{\sigma}^2) - \frac{\mu_n^2 + \sigma_n^2}{2\overline{\sigma}^2} + \frac{2\mu_n\overline{\mu}}{2\overline{\sigma}^2} - \frac{\overline{\mu}^2}{2\overline{\sigma}^2} \\
&= -\frac{1}{2}\log(2\pi\overline{\sigma}^2) - \frac{\sigma_n^2 + (\mu_n - \overline{\mu})^2}{2\overline{\sigma}^2}.
\end{aligned}$$

Then:

$$\int q(t|x_n, s_n) \log q(t|x_n, s_n) \mathrm{d}t = \int \frac{e^{-\frac{(t-\mu_n)^2}{2\sigma_n{}^2}}}{\sqrt{2\pi}\sigma_n} \log \frac{e^{-\frac{(t-\mu_n)^2}{2\sigma_n{}^2}}}{\sqrt{2\pi}\sigma_n} \mathrm{d}t$$
$$= -\frac{1}{2} - \frac{1}{2} \log(2\pi\sigma_n^2).$$

Finally:

$$\mathbb{KL}(q(t|x_n, s_n)||p(t)) = \int q(t|x_n, s_n)(q(t|x_n, s_n) - \log p(t)) \mathrm{d}t$$
$$= -\frac{1}{2} + \log \frac{\overline{\sigma}}{\sigma_n} + \frac{\sigma_n^2 + (\mu_n - \overline{\mu})^2}{2\overline{\sigma}^2}. \quad \text{(S5)}$$

Supplementary Note 4: Alignment on technical variability

We consider three types of technical variability of gene expression for each cell: dropout events, batch effects and different sequencing depth, as quantified by library size. Since our method is based on variational autoencoder, we can deduce the form of gene expression after aligning on dropout events, batch effects, and different sequencing depth or combinations thereof for each cell. The most popularly researched alignment involves dropout events for each cell and each gene, which we term as imputation. In scVIC, given the measured gene expression x_n, batch annotation s_n and the trained neural networks, the gene expression of the $n-$th cell can be imputed as y_n^{Im} by

$$\begin{aligned} z_n^{\text{Encoder}} &= f_{\mu^z}(x_n, s_n), \\ t_n^{\text{Encoder}} &= f_{\mu^t}(x_n, s_n), \\ y_n^{\text{Im}} &= t_n^{\text{Encoder}} f_\rho(z_n^{\text{Encoder}}, s_n). \end{aligned}$$

In the context of autoencoder, z_n^{Encoder} and t_n^{Encoder} are the mean of latent variables z_n and t_n encoded from x_n and s_n through the trained neural networks, and y_n^{Im} is the gene expression decoded from z_n^{Encoder} and t_n^{Encoder} before dropout events take place.

Another popularly researched alignment involves batch effects for each cell and each gene, which we term as calibration. In scVIC, given the measured gene expression x_n, batch annotation s_n and the trained neural networks, we can calibrate the $n-$th cell to y_n^{Ca} on the given reference batch $\overline{s}$ as

$$\begin{aligned} z_n^{\text{Encoder}} &= f_{\mu^z}(x_n, s_n), \\ t_n^{\text{Encoder}} &= f_{\mu^t}(x_n, s_n), \\ y_n^{\text{Ca}} &= t_n^{\text{Encoder}} f_\rho(z_n^{\text{Encoder}}, \overline{s}). \end{aligned}$$

Compared to achieving gene expression y_n^{Im} based on s_n and decoded from z_n^{Encoder} and t_n^{Encoder} on imputation, calibration achieves gene expression y_n^{Ca} transferred from original batch s_n to the given reference batch $\overline{s}$, as $f_\rho(z_n^{\text{Ca}}, \overline{s})$ shows. Obviously, calibration also returns the gene expression before dropout events take place.

The remaining alignment involves different sequencing depth for each cell, and, though it is rarely researched, we term it as registration. In scVIC, given the measured gene expression

x_n, batch annotation s_n and the trained neural networks, we can register the $n-$th cell to expected expression proportion ρ_n^{Re} on the given reference batch $\overline{s}$ as

$$\begin{aligned} z_n^{\text{Encoder}} &= f_{\mu^z}(x_n, s_n), \\ \rho_n^{\text{Re}} &= f_\rho(z_n^{\text{Encoder}}, \overline{s}), \end{aligned}$$

where ρ_n^{Re} achieves proportional transcription expression across genes by eliminating scaling factor $t_n$of cell n. Registration also eliminates dropout events and batch effects. The value of registration is comparable across cells suffering from dropout events, batch effects and different sequencing depth, and can be taken as a standard value that reflects biological information free from all three types of technical variability.

Supplementary Note 5: Differential expression

For each gene g and each component c^* of GMM, we can formulate two mutually exclusive hypotheses:

$$H_1^g := \mathbb{E}_s f^g_{\rho|z,s}(z_{c^*}, s) > \mathbb{E}_s f^g_{\rho|z,s}(z_{\overline{c}}, s),$$
$$H_2^g := \mathbb{E}_s f^g_{\rho|z,s}(z_{c^*}, s) \leq \mathbb{E}_s f^g_{\rho|z,s}(z_{\overline{c}}, s).$$

H_1^g assumes that the expression level of gene g in component c^* is greater than its expression levels in the remaining components, while H_2^g assumes that the expression level of gene g in component c^* is less than its expression levels in the remaining components. Expectation $\mathbb{E}_s$ is calculated across empirical frequencies of different batches, z_{c^*} denotes latent variables inclusive to component c^*, and $z_{\overline{c}}$ denotes latent variables exclusive to component c^*. It is worth noting that network $f_{\rho|z,s}$ decodes the mean proportion of transcriptional expression across genes, eliminating the scaling factor of different cells and dropout events. We can use a Bayes factor to evaluate whether H_1^g or H_2^g is more probable:

$$K = \log_e \frac{p(H_1^g|c^*)}{p(H_2^g|c^*)}.$$

Sign of the Bayes factor indicates which hypothesis is more likely and magnitude of the Bayes factor reveals the significance level. Throughout the paper if $|K| > 3$ (equivalent to an odds ratio of $\exp(3) \approx 20$), then we consider a Bayes factor strong enough to favor one hypothesis over the other. The specific density function of H_1^g is defined below and that of H_2^g is similar.

$$p(H_1^g|c^*) \approx \sum_s \sum_{\overline{c} \neq c*} \iint_{z_{c^*}, z_{\overline{c}}} P_1(z_{c^*}, z_{\overline{c}}|c^*, \overline{c}, s) p(\overline{c}) p(s) \mathrm{d}z_{c^*} \mathrm{d}z_{\overline{c}},$$
$$P_1(z_{c^*}, z_{\overline{c}}|c^*, \overline{c}, s) = \mathbb{I}[f^g_{\rho|z,s}(z_{c^*}, s) > f^g_{\rho|z,s}(z_{\overline{c}}, s)] \mathcal{N}(z_{c^*}; \mu_{c^*}, I) \mathcal{N}(z_{\overline{c}}; \mu_{\overline{c}}, I),$$

where $\mathbb{I}$ denotes indicator function, $p(s)$ designates the relative abundance of cells in batch s and $p(\overline{c})$ represents the proportion of the $\overline{c}-$th component other than the c^*-th component, which is dependent on $[\pi_c]_C$. We can use naive Monte Carlo to compute these integrals.

Supplementary Note 6: Differential expression on the RETINA dataset

In addition to capturing the heterogeneity of the RETINA datasets, we further conducted a differential expression analysis on this dataset. Fig.S4A shows the clustering results of scVIC, which is directly derived solely from the intrinsic GMM model. It revealed 11 distinct clusters. Compared to the clustering results obtained using scVIC-Louvain, scVIC merged several finely subdivided sub-clusters into a larger clusters, notably clusters 0, 7, 8, 11, and 14. This amalgamation may stem from the linear nature of the GMM employed, wherein the clusters of these finely divided sub-clusters exhibits significantly smaller inter-class distances.

After the training of scVIC is completed, differential expression analysis was performed based on Supplementary Note 5. It is worth noting that this export is not strictly post-hoc. Traditional differential expression analysis is based on pre-clustered results, re-examining the differences in gene expression between cells within each cluster and other clusters. The built-in differential expression used in scVIC is directly based on the trained model. scVIC establishes hypothesis testing based on an optimized probability generation process, without reusing gene expression of each cell. The results of scVIC's differential expression analysis are shown in Fig.S4B, where genes with Bayes factors $K > 3$ were selected for each cluster to filter highly differentially expressed genes, showcasing the top 2. The rows of the matrix plot represent the index of highly differentially expressed genes (on the left) and their corresponding clusters (on the right), while the columns denote the index of clusters. The value of the matrix plot is taken as the raw gene expression. It can be observed that the mean expression correspond well to the differential expression genes derived from scVIC (the highly differentially expressed genes exported by different clusters are indeed differentially expressed), which means that scVIC has effectively incorporated the heterogeneity presented by differential expression into the model.

We further compared the variable ρ (the registration variable referenced in Supplementary Note 4, which is free from technical variability and corresponds to the mean of proportional transcription expression across genes in the encoder of the scVIC model, with raw gene expression. The specific expression differences of highly differentially expressed genes between cells are showed in Fig.S4C. Overall, ρ was found to more clearly differentiate between different clusters (although there are expression differences, ρ shows higher contrast). For some ambiguous genes in terms of raw gene expression differences, ρ clearly showed differential expression, such as gene index 10,098 in cluster 3, gene index 4,119 in

cluster 4, and gene index 11,156, 2,521 in cluster 8, and gene index 1,624 and 1,839 in cluster 14.

Supplementary Figures

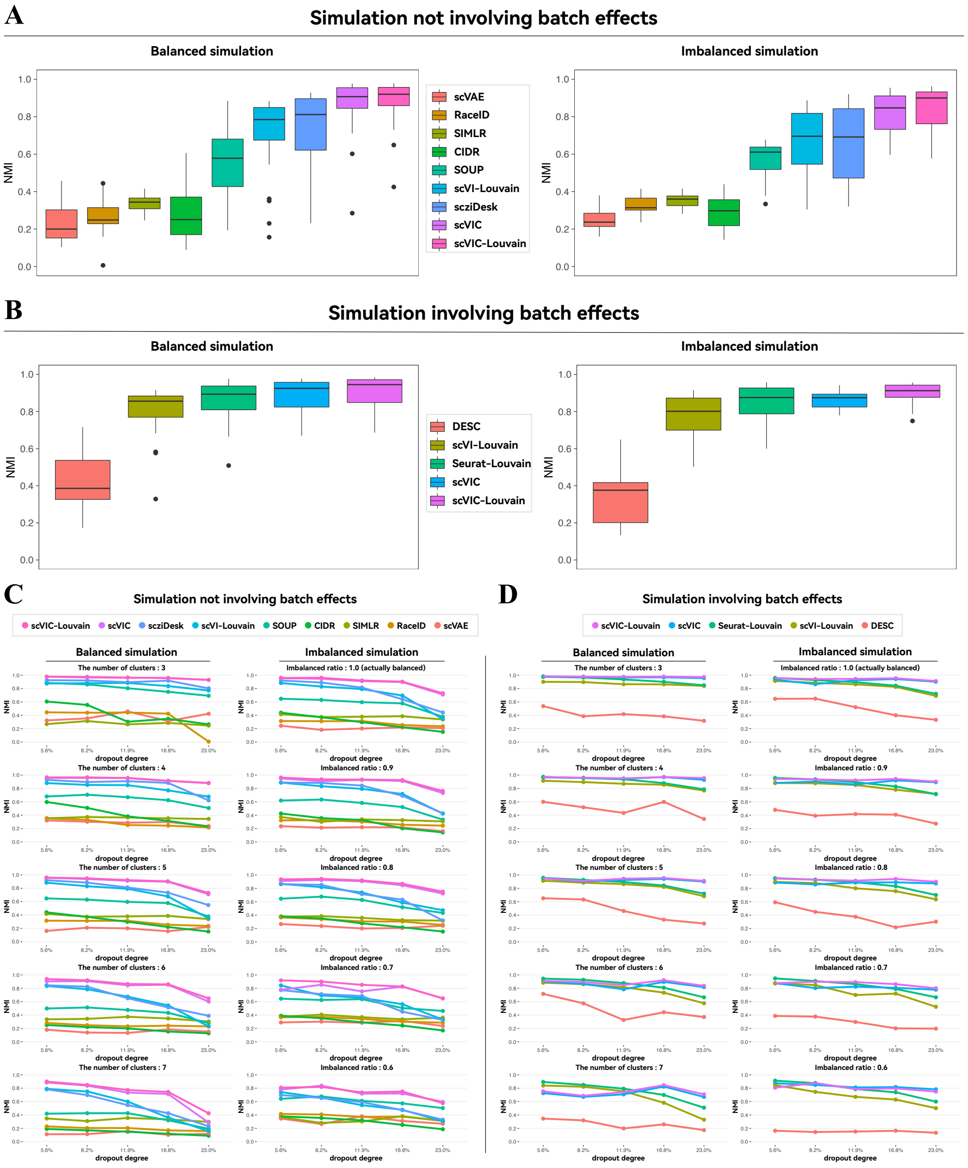

Supplementary Figure S1. Comparison (NMI) of clustering algorithms on simulation datasets. Bar charts of NMI for different clustering algorithms on simulation datasets **(A)** not involving batch effects and **(B)** involving batch effects. For different simulation parameters, line charts of NMI for different clustering algorithms on simulation datasets **(C)** not involving batch effects and **(D)** involving batch effects. The source data used in this figure is placed in the Supplementary Tables.

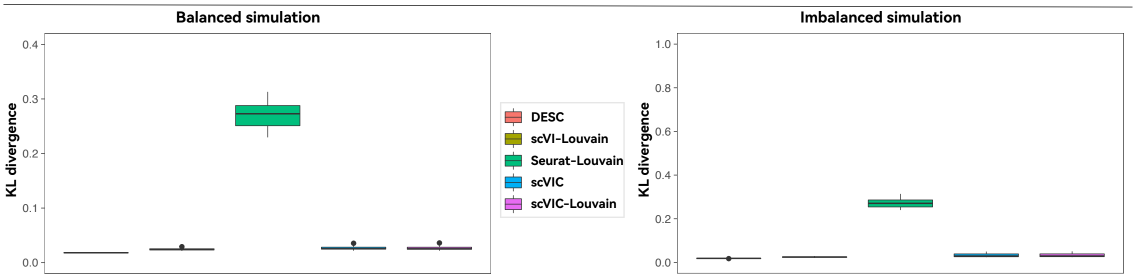

Supplementary Figure S2. Bar charts of KL divergence for different algorithms on simulation datasets involving batch effects. The designed KL divergence is used to measure the removal performance of batch effects where smaller indicates better. The source data used in this figure is placed in the Supplementary Tables.

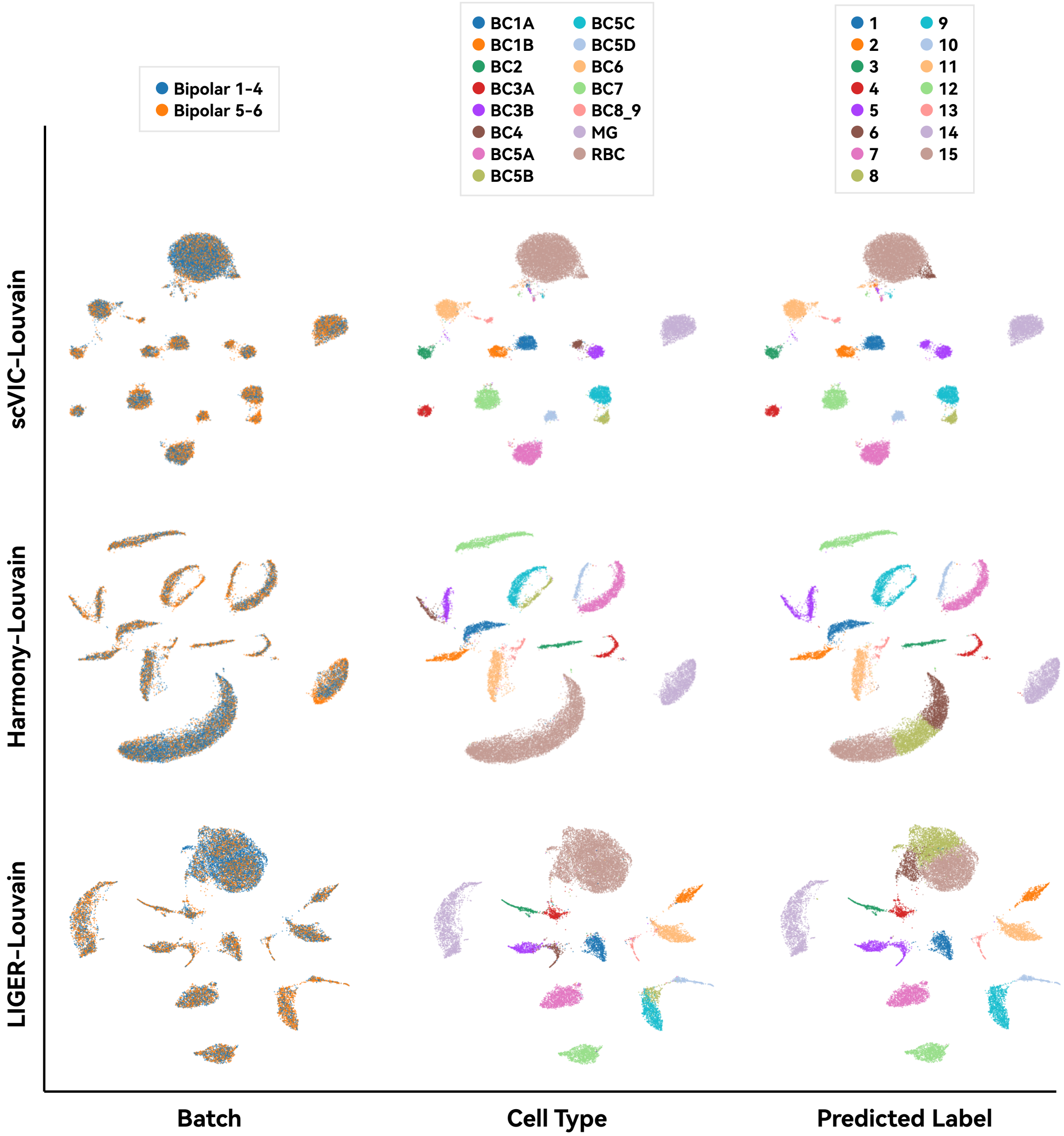

RETINA

	Entropy of Batch Mixing	ARI	NMI
**scVIC-Louvain**	0.05531	0.94491	0.94215
**Harmony-Louvain**	0.07634	0.57234	0.85776
**LIGER-Louvain**	0.04691	0.61911	0.85327

Supplementary Figure S3. Comparison of scVIC, Harmony, and LIGER on the RETINA dataset.

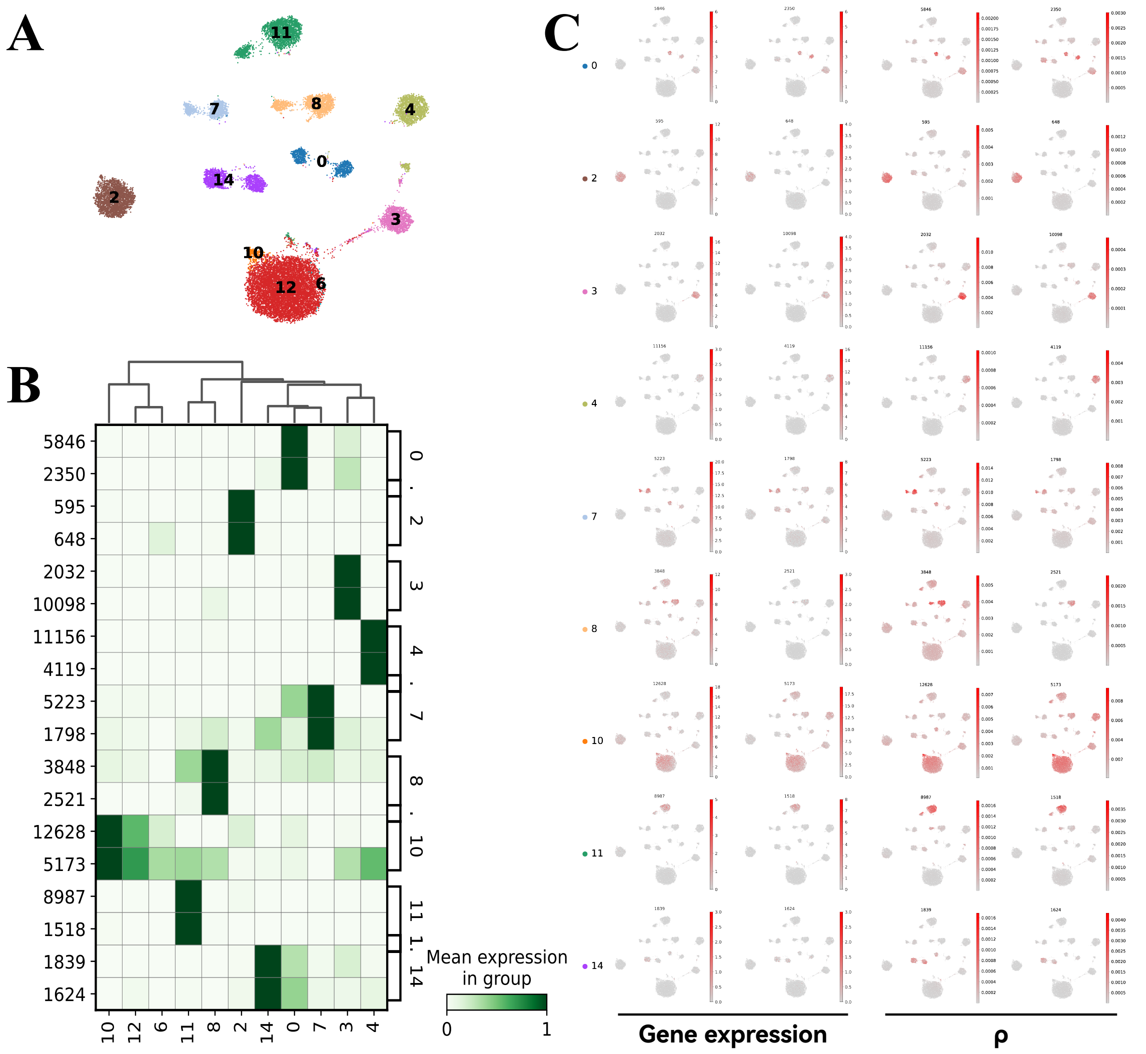

Supplementary Figure S4. Differential expression analysis on the RETINA dataset. **(A)** Clustering results of scVIC. **(B)** Matrix plot of mean gene expression of highly differentially expressed genes. The rows of the matrix represent the index of highly differentially expressed genes (on the left) and their corresponding clusters (on the right), while the columns denote the index of clusters. The value of the matrix is taken as the raw gene expression. **(C)** Dot plot of gene expression of highly differentially expressed genes.
